# Overview of the meningeal lymphatic vessels in aging and central nervous system disorders

**DOI:** 10.1186/s13578-022-00942-z

**Published:** 2022-12-17

**Authors:** Huimin Jiang, Huimin Wei, Yifan Zhou, Xuechun Xiao, Chen Zhou, Xunming Ji

**Affiliations:** 1 Beijing Institute of Brain Disorders, Laboratory of Brain Disorders, Ministry of Science and Technology, Collaborative Innovation Center for Brain Disorders, Beijing Advanced Innovation Center for Big Data-Based Precision Medicine, Capital Medical University, Beijing, 100069 China; 2 Beijing Advanced Innovation Center for Big Data-Based Precision Medicine, School of Biological Science and Medical Engineering, Beihang University, Beijing, 100191 China; 3 Department of Neurosurgery, Xuanwu Hospital, Capital Medical University, Beijing, 100053 China

**Keywords:** Meningeal lymphatic vessels, Aging, Neurological disorders, Lymphatic drainage, Central nervous system

## Abstract

In the aging process and central nervous system (CNS) diseases, the functions of the meningeal lymphatic vessels (MLVs) are impaired. Alterations in MLVs have been observed in aging-related neurodegenerative diseases, brain tumors, and even cerebrovascular disease. These findings reveal a new perspective on aging and CNS disorders and provide a promising therapeutic target. Additionally, recent neuropathological studies have shown that MLVs exchange soluble components between the cerebrospinal fluid (CSF) and interstitial fluid (ISF) and drain metabolites, cellular debris, misfolded proteins, and immune cells from the CSF into the deep cervical lymph nodes (dCLNs), directly connecting the brain with the peripheral circulation. Impairment and dysfunction of meningeal lymphatics can lead to the accumulation of toxic proteins in the brain, exacerbating the progression of neurological disorders. However, for many CNS diseases, the causal relationship between MLVs and neuropathological changes is not fully clear. Here, after a brief historical retrospection, we review recent discoveries about the hallmarks of MLVs and their roles in the aging and CNS diseases, as well as potential therapeutic targets for the treatment of neurologic diseases.

## Introduction

Aging is an important cause of CNS diseases and even affects the prognosis of these diseases, including cerebrovascular diseases, neurodegenerative diseases, trauma, brain tumors and so on. It has long been thought that the pathogenic effect of aging is the direct senescence of neurons, and the progress of immunology has emphasized the immune interactions between the CNS and periphery [1]. The CNS has been considered a relatively immune-privileged site. While the neuroimmune interactions play an important role in diverse neurological disorders, immune surveillance of the CNS remains unclear. The CNS contains microglia, but these cells are confined to the brain parenchyma and cannot interact with peripheral immune system under healthy conditions [2]. Unlike the brain parenchyma, the meningeal lymphatic network enables immune surveillance of the brain efficiently. The discovery of MLVs in the CNS has shattered the traditional notion that the CNS is immune-privileged [3, 4]. Aging is accompanied by a functional decline of MLVs, which contribute to several age-related neurodegenerative diseases, such as Alzheimer’s disease (AD), Parkinson’s disease (PD), brain tumors, traumatic brain injury (TBI), multiple sclerosis (MS), and stroke [5–11].

Over the past few years, evidence for MLVs in the CNS has been accumulating. Recent studies revealed that some features of the meningeal lymphatic system are also present in humans [12, 13]. Defects in MLVs, which excrete metabolic wastes from the CNS to peripheral surroundings, are implicated in various neurological disorders. Although the contribution of MLVs in these diseases is not completely understood, the accumulation of metabolites, cellular debris, and misfolded proteins in the brain due to impaired drainage, which cannot be transported to dCLNs, may play key roles [14, 15]. It has been gradually recognized that the CNS relies on the function of MLVs to maintain homeostasis, and the draining function of MLVs also decreases with age [14].

In this review, we summarize the basic concepts of the history of MLVs briefly and then introduce the development and function of the meningeal lymphatics. Finally, we discuss the relationship between MLVs and neurological disease.

## The meningeal lymphatic vessels—a forgotten chapter of human anatomy

**Fig. 1:**
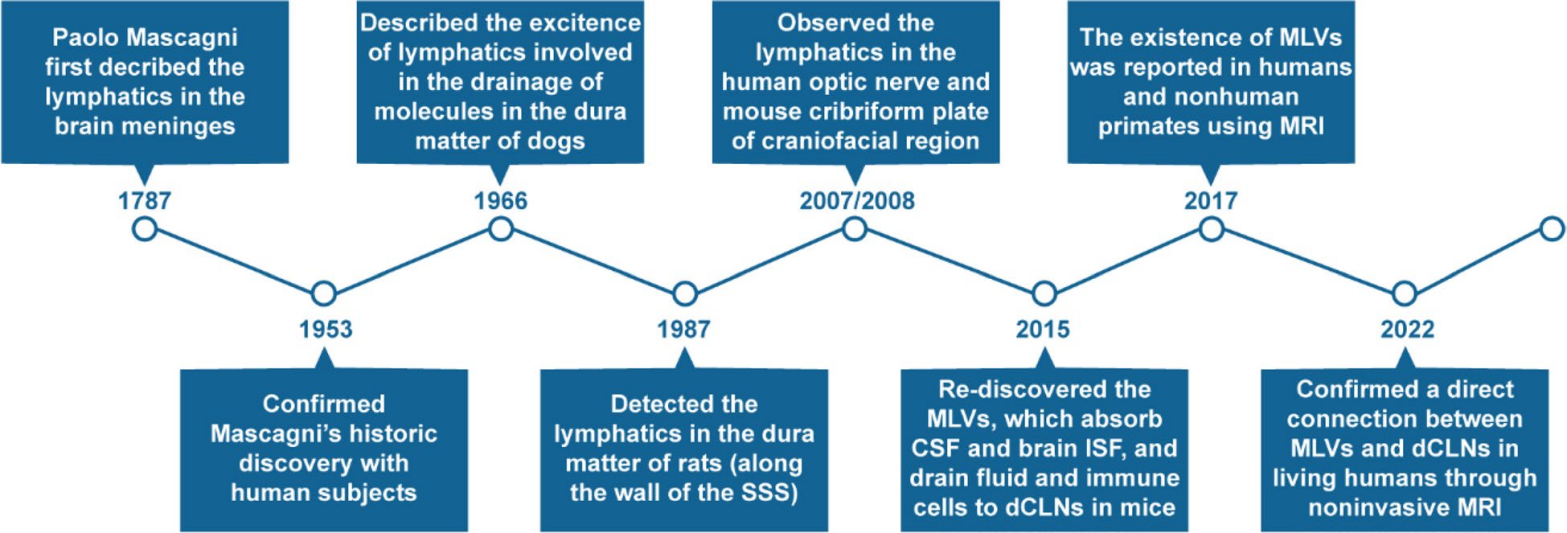
A brief history of the (re)discovery and development of MLVs

In the late eighteenth century, the anatomist Paolo Mascagni firstly described the presence of lymphatics in the cerebral dura mater [16, 17]. However, for a long time after that, that description of lymphatic vessels and even the relevant chapter of human anatomy were forgotten. It was not until 1953 that Lecco confirmed this historic view in his studies with human subjects [18]. Foldi et al. observed the presence of lymphatic vessels in the dura matter of the skull base by injection of Congo red solution and showed that these lymphatic vessels were connected to the cervical lymph trunk in dogs in 1966 [19]. Incidentally, lymphatic vessels were also detected along the wall of the superior sagittal sinus (SSS) in the dura mater of rats during an electron microscopy study in 1987 [20]. It has been verified that there are lymphatic vessels in the human optic nerve through immunostaining for the molecular marker D2-40 [21]. Furthermore, the lymphatic system observed on the mouse cribriform plate in the craniofacial region by electron microscopy was the route for CSF transport [22]. According to classical theories, CSF is produced by the choroid plexus of the lateral ventricle, third ventricle and fourth ventricle and then absorbed by arachnoid granulations into the cerebral venous sinuses [23]. However, several studies have demonstrated that the brain uses the “glymphatic” system to clear macromolecules in the CSF and ISF, similar to the functions of lymphatic vessels [24–26]. Eventually, Aspelund and Louveau et al. clearly proved that the meningeal lymphatic vasculature in the dura mater of the CNS in mice can absorb CSF from the adjacent subarachnoid space (SAS) and brain ISF via the “glymphatic” system [3, 4]. They also demonstrated that the MLVs had the ability to carry fluid and immune cells to the dCLNs. Recently, the existence of MLVs was reported in both humans and nonhuman primates using in vivo magnetic resonance imaging (MRI) techniques and confocal microscopy [13, 27–29]. Importantly, Albayram et al. showed a direct connection between MLVs and dCLNs in living humans through noninvasive MRI [12] (Fig. 1). Therefore, the existence of MLVs has been recognized by most scholars. Studying the functions of MLVs may provide new approaches for the pathogenesis, early diagnosis, and therapy of CNS disease.

## The development and structure of MLVs

### The development of MLVs and lymphangiogenesis

Understanding of the molecular mechanisms that control lymphangiogenesis has improved in recent years due to the progress in the identification of regulatory molecules and specific markers of lymphatic endothelial cells (LECs). LECs are terminally differentiated cells derived from venous endothelial cells, and lymphatic development begins at approximately 6 to 7 weeks of embryonic development in humans and 9.5 to 10.5 days in mice [30]. Venous endothelial cells, the LEC precursors, express the homeobox transcription factor Sox18 at embryonic day (E) 9.0, and Sox18 then activates the transcription of Proprospero homeobox protein 1 (Prox1) by binding to its promoter, after which the lymphatic vessels begin to develop [31, 32]. Targeting of the Sox18 locus or homozygous mutation of Sox18 abolished the expression of Prox1 and resulted in fatal embryonic edema. Importantly, loss of one allele or heterozygous mutation of Sox18 led to defects in cutaneous lymphatic vessels, suggesting that Sox18 is necessary for the LECs differentiation program [33].

Prox1 is a specific marker of LECs in the lymphatic vascular system. In mice, Prox1-positive endothelial cells are first detected in the lymphatic endothelial hyaluronic acid receptor 1 (Lyve-1)-positive cell subpopulation on one side of the anterior cardinal vein at E9.5, and thereafter, Prox1 induces the expression of LEC-specific genes and inhibits the expression of blood endothelial cell-specific genes by binding to the nuclear receptors COUP-TFII, ultimately driving the acquisition and maintenance of lymphatic properties [34–36]. Prox1-negative endothelial cells fail to express LEC markers and instead retain their blood vascular endothelial properties [37], which further shows that Prox1 functions as a regulator that determines LECs fate.

Lyve-1 increases the expression of podoplanin (PDPN), which induces platelet aggregation. The expression of PDPN is detected in LECs at E12.5 [31]. In addition, VEGFR-3 was one of the first LECs markers to be discovered; VEGFR-3 can be activated by VEGF-C and initiates the sprouting of LECs [38]. Homozygous deletion of VEGF-C results in the absence of the mouse embryonic lymphatic vascular system, while VEGF-C heterozygous mice suffer severe lymphatic hypoplasia, indicating that VEGF-C is essential for lymphangiogenesis [38]. Additionally, although fibroblast growth factor 2 (FGF-2), insulin-like growth factor 1 (IGF-1), IGF-2, hepatocyte growth factor (HGF), endothelin-1 (ET-1), and PDGF-B have been reported to induce lymphangiogenesis, most of these effects may be secondary to the induction of VEGF-C or VEGF-D [39].

At approximately E14.5, the lymphatic development stage is completed, and Sox18 expression begins to decrease and is not involved in maintaining lymphatic identity [40].

**Fig. 2:**
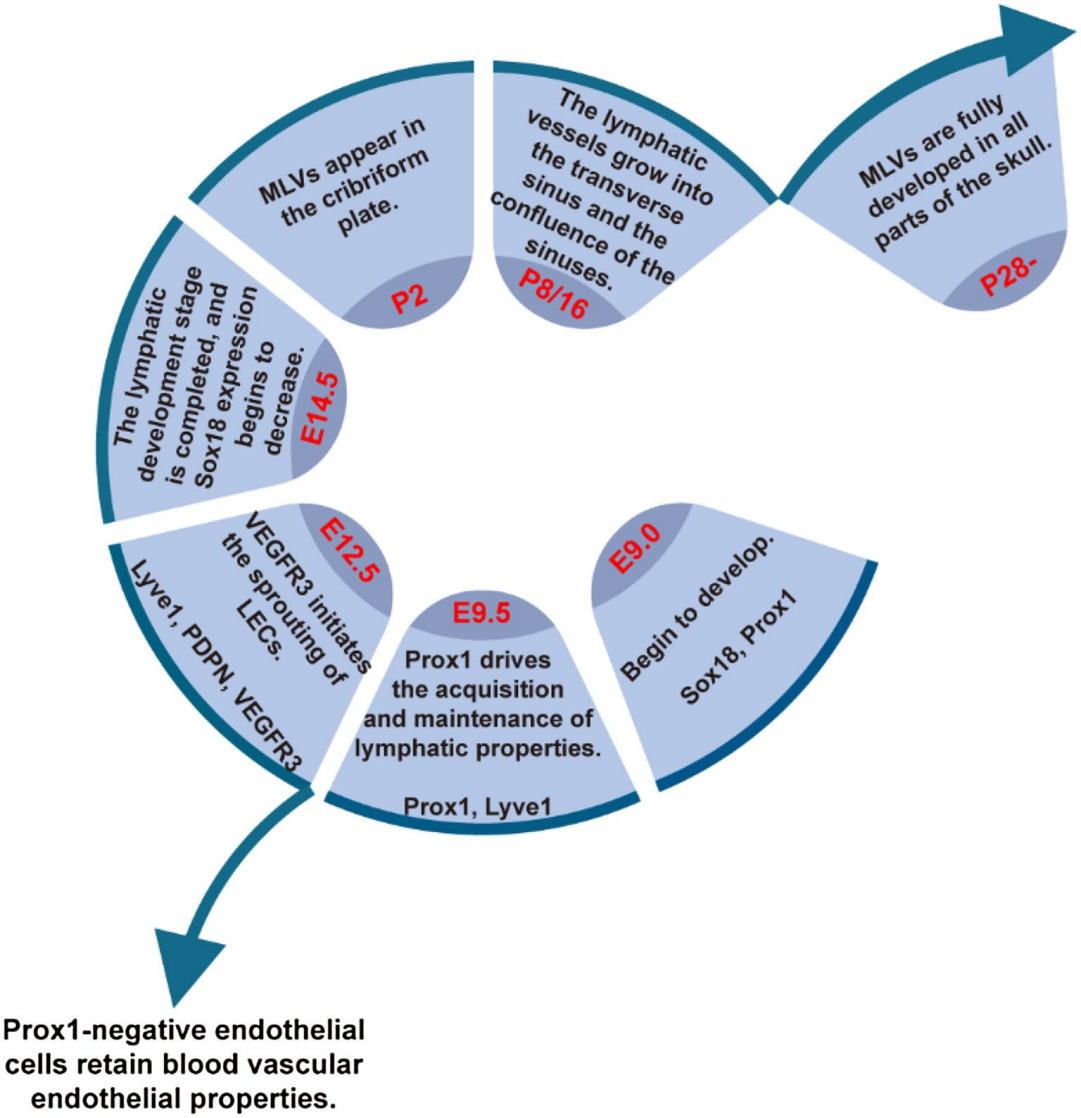
The development of MLVs

In the first month of life, MLVs appear at the base of the skull before birth and in the cribriform plate at postnatal day 2 (P2). The sprouts reach the middle meningeal artery after P4. Subsequently, the lymphatic vessels grow into the transverse sinus (TS) at P8 and the confluence of the sinuses at P16. In the end, the MLVs cover the SSS, medial meningeal artery, and rostral rhinal vein, with all parts of the skull fully developed at P28 [41–43] (Fig. 2).

In adults, lymphangiogenesis occurs primarily through the sprouting of LECs in preexisting lymphatic vessels, and it can only occur under pathological conditions such as inflammation, tissue repair, and tumor growth, all of which are induced by VEGF-C [30, 44, 45]. Nevertheless, inhibition of VEGFR3 signaling leads to degeneration of MLVs [41].

### The structure of MLVs

The lymphatics consist of a highly permeable initial lymphatic vessels and larger collecting lymphatics. Initial lymphatic vessels are thin-walled vessels formed by a single layer of LECs that respond to VEGF-C to activate lymphangiogenesis [15, 46]. In addition, initial lymphatic vessels are linked in small button-shaped junctions with the presence of discontinuous basement membranes and lack smooth muscle cells (SMCs). The button junctions and anchoring filaments construct the primary lymphatic valves that permit the entry of ISF, macromolecules and immune cells [30, 47]. Subsequently, the initial lymphatic vessels gradually drain into pre-collecting and collecting lymphatics, which are larger in caliber and surrounded by SMCs. Collecting lymphatics consist of tightly continuous zipper-like connections and secondary intraluminal valves that are virtually impermeable to prevent lymphatic backflow and facilitate unidirectional drainage [48]. LECs within initial lymphatic vessels are anchored to the extracellular matrix, but the molecular mechanisms that regulate the association of LECs with the extracellular matrix are largely unknown. Emilin1, an elastic microfibril-associated protein, affects LEC anchorage, and its deficiency leads to hyperplasia of lymphatic vessels, with a reduced number of anchoring filaments, aberrant intercellular junctions and impaired lymphatic drainage function in mice [49].

Structurally, the lymphatic vessels in the meninges are patterns alongside arteries and veins, including the SSS, TS, sigmoid sinus, retroglenoid vein, rostral rhinal vein, middle meningeal artery, and pterygopalatine artery [3, 4, 42, 50]. Additionally, the meningeal lymphatic network in the TS is larger and more complex compared to the SSS [4]. The lymphatic flow in MLVs occurs in the opposite direction of the venous flow in the SSS of the human brain [13]. Lymphatic vessels are composed of LECs and MLVs express LECs surface marker proteins, including Lyve-1, Prox1, PDPN and VEGFR3, which can be used to detect lymphangiogenesis and lymphatic vessel invasion [4, 32]. Significantly, the initial MLVs in the dorsal parts aggregate into larger collecting lymphatics at the skull base, forming a lymphatic network that runs through the entire dural layer [3, 50, 51]. The collecting MLVs at the base of the skull then extend along the jugular vein, leave the skull via the various foramina, and confluence with the peripheral collecting lymphatics, which drain mainly to the dCLNs [3, 41, 50, 51].

**Fig. 3:**
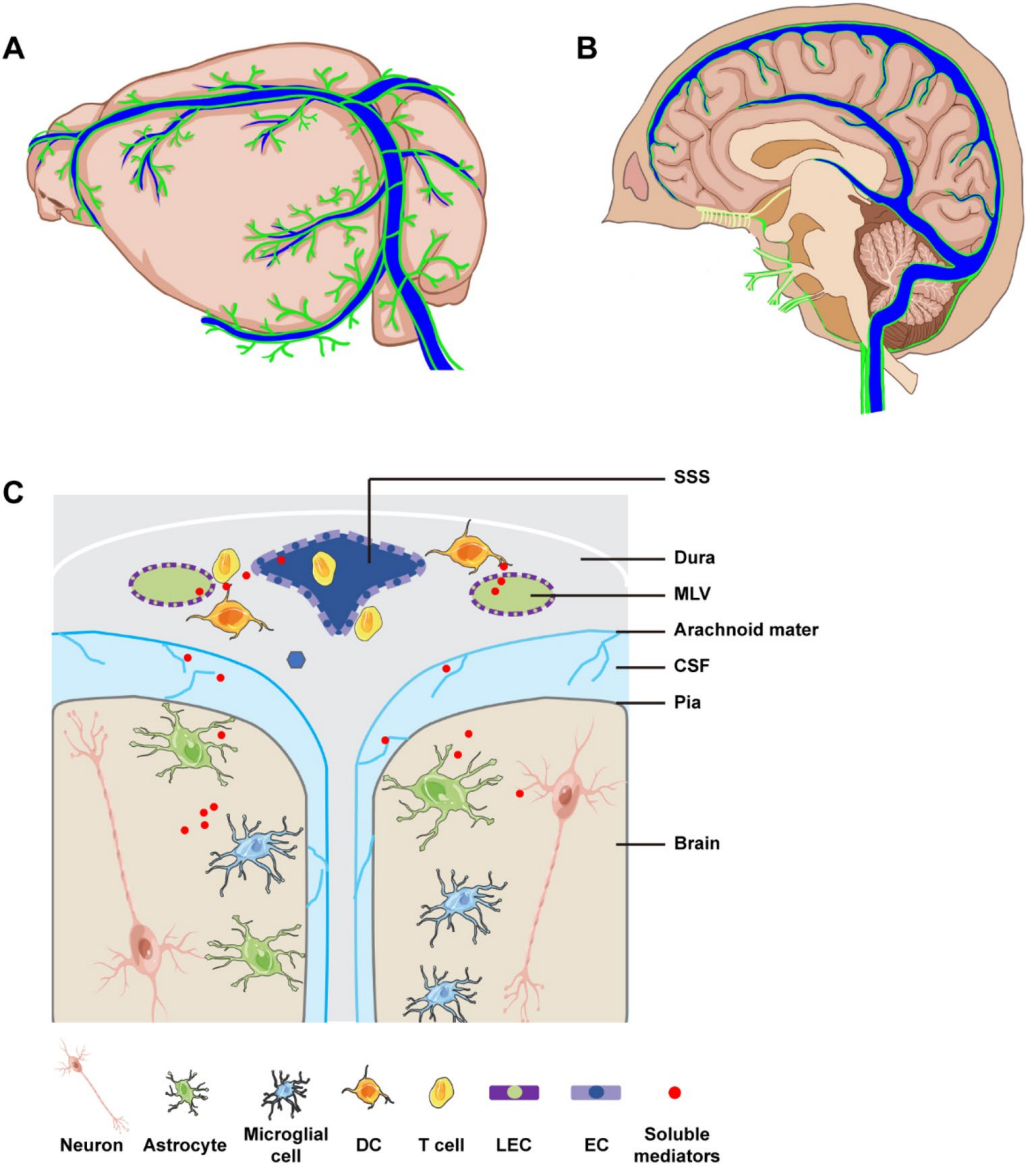
Schematic model of the meningeal lymphatic system and its drainage pathways. **A** Morphology of rodent MLVs. MLVs lie within the outer layer of the meninges and extend along the SSS to the confluence of sinuses (COS) and then further along the TS and the sigmoid sinus to join the dCLNs. **B** The dural lymphatic structures and venous system in humans. MLVs are distributed along the dural venous sinuses, draining along the jugular veins and foramen magnum after leaving the skull, and along the cranial nerves, jugular veins and internal carotid artery in the neck and connecting with the cervical lymph nodes. **C** Schematic representation of the connections between MLVs and other brain cells and CSF. The MLVs act as an important efflux pathway that drains antigens, immune cells, and CSF to dCLNs. Cytokines secreted by stromal cells in MLVs microenvironment play a key role in regulating the structure and function of MLVs

Louveau et al. firstly demonstrated that Lyve-1-positive MLVs are located near the dural sinuses and exhibit a conduit-like structure. There are two to three MLVs expressing Lyve-1 on either side of the dural sinus and parallel to the dural sinus [4]. At the same time, Aspelund and colleagues verified that Prox1-posotive MLVs were adjacent to the dural blood vasculature [3] (Fig. 3A).

Most of our knowledge of MLVs comes from animal studies, but comparable data from human studies are limited. In recent years, the existence of MLVs has been visualized by MRI in human and nonhuman primates [13, 27]. Using MRI, Albayram et al. described in detail the dorsal distribution of MLVs along the venous sinuses and the direct relationship between MLVs and dCLNs; specifically, MLVs were apparent around almost all dural venous-parasagittal structures and served as a continuous fluid channel from the base of the skull to the dCLNs in humans [12] (Fig. 3B). Although lymphatic vessels or LECs were discovered in the cribriform plate and basal skull in recent years, their functions in neurologic diseases have rarely been reported. Instead, the MLVs in the dorsal part of the skull have been shown to be involved in the progression of many neurodegenerative disorders [52].

### The remodeling of the MLVs

Under physiological and pathological conditions, the structure and function of MLVs are remodeled by the microenvironment, in which cytokines play a key regulatory role. Recent work has found that some soluble mediators, such as interleukin and VEGF-C mediate the remodeling of lymphatic vessel structure and function [53]. In CNS diseases, astrocytes [54, 55], vascular endothelial cells [14, 51], and immune cells [2, 56, 57] can secrete the above factors and participate in the pathological remodeling of the MLVs.

Under homeostasis, the dural meninges are infiltrated by numerous immune cells (except microglia), which exert immune surveillance and affect brain function [58]. Immunofluorescence labeling of CD3 and MHCII showed that T cells and MHCII^+^ antigen-presenting cells (APCs) were present in high concentrations near the dural sinuses (perisinusal), mainly restricted to the meningeal space [2, 4]. APCs effectively present antigens to T cells by expressing MHCII. APCs comprise resident immune cells, like microglia, astrocytes, peripheral macrophages and dendritic cells (DCs), which migrate from the brain parenchyma to the lymph nodes via the meningeal lymphatic system [59]. Hence, MLVs provide a physical connection for antigens and immune cells from the CSF to enter the draining lymph nodes and promote the priming and activation of T cells (Fig. 3C).

Nevertheless, Louveau et al. reported that disruption of MLVs function would inhibit the priming of T cells in dCLNs, T cells and DCs injected into CSF can be detected in the MLVs that further drain them to the dCLNs [51]. Ablation of meningeal lymphatics could block the migration pathway of immune cells and reduce the activation of T cells in dCLNs, thereby reducing the severity of experimental autoimmune encephalomyelitis (EAE) [51]. Notably, the integrity of the MLVs is indispensable for antitumor immunotherapy. For example, VEGF-C activated tumor-specific T cells by promoting lymphangiogenesis in glioblastoma multiforme (GBM), which increased the anti-tumor effects of anti-PD-1 and prolonged the survival of mice [60]. In addition to T cells and DCs mentioned above, the presence of other leukocytes, such as macrophages, was observed adjacent to the MLVs when neuroinflammation occurred, suggesting that macrophages may be recruited through the meningeal lymphatics [2, 10].

As for the relationship between MLVs and blood vessels, meningeal LECs lack astrocytic end-feet and differ from brain endothelial cells in many other ways. Thus, the blood-meningeal barrier allows immune cells to enter more than the blood–brain barrier [61]. Claudin-5 and VE-cadherin showed a continuous expression pattern in the blood vessels, whereas a punctate pattern was observed in MLVs. Intracerebroventricularly injection with tracer dye (QDot655) demonstrated that the QDot655-filled MLVs had a slower rate of flow than the adjacent blood vessels [4].

In conclusion, meningeal LECs are important stromal cell types in the CNS that are regulated by the MLVs microenvironment and are also involved in the remodeling of the microenvironment. Therefore, exploring the relevant regulatory relationships between meningeal LECs and other meningeal cells is a new perspective for understanding the pathological processes of many CNS diseases. Enhancement of meningeal lymphatic function by VEGF-C has been shown to be protective against brain tumors [60] and TBI [8] mice, also suggesting that MLVs are a valuable target for intervention in many CNS diseases.

## Multifunctional characterization of the meningeal lymphatics

The newly recognized presence of functional MLVs in the CNS has shed new light on the etiology of a variety of neurological disorders. In recent years, it has been reported that MLVs have important impacts on the complex circulation and exchange of soluble contents between the CSF and ISF [62].

### Meningeal lymphatics—the “guardian” of CSF/ISF homeostasis

The total volume of CSF is renewed approximately 11 times a day in rats and approximately 4 times a day in healthy humans. The fluid in the CNS outflows mainly through three pathways: arachnoid granulations and villi in the dural venous sinus [63], peripheral lymphatic vessels near the cribriform plate [22], and MLVs [3, 4]. When measuring the proportion of CSF drained from the lymphatic system with deep cervical lymphatic cannula and radioactive tracers in rabbits and sheep, the lymphatic vessels account for approximately 30–50% of the total outflow [64]. More importantly, recent studies have shown that the main route of CSF outflow is through MLVs rather than the dural venous sinus and nasal lymphatics, highlighting the importance of this drainage pathway for the clearance of molecules in the brain [50]. MLVs function as channels to carry macromolecules, antigens, immune cells and fluid from the CSF toward the dCLNs [3, 4, 51].

In addition, MLVs and the glymphatic system are the main routes to clear waste from the brain [65, 66]. The term “glymphatic” pathway was first proposed by Nedergarrd’s team. This system is dependent on glial water flux and lymphatic function for interstitial solute clearance [24]. The glymphatic system drives CSF influx into the brain parenchyma along the peri-arterial space. Aquaporin-4 (AQP-4) is expressed in vascular astrocytic endfeet and promotes glymphatic transport and the mixing of CSF with ISF [67]. Mesquita et al. reported that functional MLVs regulated the normal route of the glymphatic system, and there was a direct link between MLVs and the glymphatic system via CSF/ISF. Disruption of the drainage of MLVs impaired the influx of CSF through the paravascular route (glymphatic system) and the paravascular efflux of CSF/ISF macromolecules [14]. Intraparenchymally or intracerebroventricularly injected fluorescent tracers were drained into the dCLNs; notably, the tracers were also distributed in the glymphatic system [3]. Furthermore, ligation of the cervical lymphatic vessels increased the aggregation of tracers in the MLVs and reduced tracer retention in the dCLNs [68–70]. Therefore, there is a connection between the two systems.

In the steady state, various immune cells, but not microglia, accumulate in the meningeal space to perform immune surveillance; these immune cells include T cells, macrophages, neutrophils, DCs and B cells [71]. The immune cells in the meningeal space produce cytokines in the CSF, which diffuse into the brain parenchyma through the glymphatic system and interact with neurons or glial cells [62].

In general, MLVs and the glymphatic system work together to regulate CSF/ISF homeostasis. However, the structural and functional connection between MLVs and the glymphatic system should also be studied further.

### Meningeal lymphatics—the “trapway” of the CNS

The paravascular influx/efflux of the CSF and ISF was proposed as the route of metabolites, misfolded proteins, and waste products exchange. Briefly, the subarachnoid CSF flows into the brain parenchyma along the periarterial Virchow-Robin space (influx), exchanges with the ISF, and then carries metabolites, misfolded proteins, and other waste products to flow out along the perivenous space back into the CSF (efflux). In addition, these molecules can enter or leave the brain parenchyma through the endfeet clefts of the glia limitans. Finally, CSF enters the dura mater, and the waste is drained by MLVs into the dCLNs [15, 24]. Research has shown that the main route of CSF efflux in aged mice is through the lymphatic vessels, and the outflow of CSF into the lymphatics is slower than that of young mice [64]. It has been confirmed that inhibition of MLVs function greatly affects the perfusion of macromolecules in the brain in different mouse models [14, 72, 73].

The function of the lymphatics was elucidated by injecting fluorescence-conjugated macromolecules into different CNS compartments. Louveau et al. detected the presence of dye in MLVs by injecting Evans blue into the lateral ventricle of mice, and Evans blue was also drained into the dCLNs rather than the superficial cervical lymph nodes within 30 min [4]. Notably, the lymphatic vessels filled with Evans blue, which drained into the dCLNs, were located near the internal jugular vein [4]. Furthermore, ligation of the lymph vessels blocked the accumulation of the tracer in the dCLNs compared with the sham controls, leading to an increase in the diameter of MLVs [3, 4]. When A488-OVA was injected into the brain parenchyma of WT and K14-VEGFR3 mice, a mouse model lacking MLVs, the content of A488-OVA in the brain was much higher in K14-VEGFR3 mice than in WT mice [3]. In contrast, the accumulation of A488-OVA in MLVs and dCLNs was almost abrogated, suggesting that the absence of MLVs impairs the ability to clear macromolecules from the brain [3]. After ablation of MLVs, Sandro et al. found significant deposition of Aβ42-647 or OVA-647 in the brains of mice, with an accelerated decline in cognitive function in mice with AD [14].

### Meningeal lymphatics—the “watchman” of CNS inflammation and immunity

One of the principal factors supporting the idea that the CNS is an immune privileged organ is the lack of lymphatic drainage in the brain parenchyma. However, experimental evidence suggests that immune cells (T cells, B cells and DCs) accumulate in meningeal spaces under homeostatic conditions; although these immune cells (except microglia) cannot enter the brain parenchyma, MLVs carry numerous immune cells and traffic these cells out of the CNS to play a role in immune surveillance [4, 71, 74].

Under pathological conditions, DCs recognize the corresponding antigens and migrate to the dCLNs to promote the proliferation and activation of antigen-specific T cells [75]. During this process, DCs or T cells enter MLVs primarily through the CCL21-CCR7 pathway [7, 51]. The migration of T cells to the dCLNs in a CCR7-dependent manner appears to be central for the maintenance of meningeal immunity, as shown by the observation that the drainage of CCR7-KO T cells in the dCLNs was significantly less than that of CCR7-WT T cells when the two types of cells were coinjected into CSF [51]. The lung is thought to be a “licensed” site for CNS-specific T cells to acquire migratory properties that permit them to access the CNS [76]. However, research shows that the dCLNs are another “licensed” site for T-cell reactivation [51]. Additionally, impairment of MLVs disrupts the natural dynamics of T-cell circulation out of the meninges, leading to T-cell accumulation [51]. Moreover, a previous study stated that ligation of the dCLNs leads to the accumulation of CD3^+^ T cells within the brain and dura matter, activation of glial cells and neuroinflammation in APP/PS1 mice owing to the blockade of meningeal lymphatic drainage [72]. Additionally, recent work suggested that CCR7 deficiency in 5xFAD mice increases the numbers of macrophages, B cells, neutrophils, and Tregs in the meninges, together with cognitive deficits and Aβ accumulation, which can be alleviated by anti-CD25 treatment by decreasing Tregs in the meninges and dCLNs of old mice [9].

## The relationship between MLVs and neurological disorders

### Meningeal lymphatics and AD

AD is the most common type of dementia, and it is widely believed to be driven by the extracellular plaque deposition of the Aβ peptide and intracellular neurofibrillary tangles, which are composed of hyperphosphorylated tau protein [77].

#### MLVs and the drainage of Aβ

Aβ is derived from β-amyloid precursor protein (APP) via the proteolytic functions of β-secretase and γ-secretase, while α-secretase prevents its generation by cleaving it in the middle of the amyloid domain [78]. The key to the occurrence and development of AD is the imbalance between Aβ production and clearance. Extracellular Aβ in ISF is excreted into the vascular lumen or transported into the CSF circulation through the glymphatic route [79]. Therefore, restoring the clearance function of Aβ is a promising strategy for the treatment of AD [80].

Since the CSF turnover rate in healthy elderly people is half that in young people, it is thought that the CSF turnover rate may be related to the occurrence of dementia in elderly people. Reduced CSF flow would affect the composition of the CSF so that the solute clearance ability of the CSF is reduced, thus affecting the clearance of Aβ and leading to AD in elderly individuals [81].

It is worth noting that MLVs contribute to the clearance of extracellular Aβ from the brain, as studies have shown that Aβ can accumulate within these meningeal lymphatics and further drain into the dCLNs [14, 82, 83]. Nevertheless, impaired lymphatic drainage of CSF would affect Aβ clearance and thus increase the burden of Aβ in the brain [84]. The deterioration of the MLVs in 5xFAD mice was accompanied by a significant increase in Aβ deposition compared with the wild-type littermate controls [5]. Similarly, ablation of the meningeal lymphatics resulted in increased Aβ deposition in both the meninges and the brain parenchyma, which led to neuronal dysfunction and behavioral changes in a 5xFAD mouse model [14]. Likewise, ligation of lymphatic vessels afferent to the dCLNs exacerbates Aβ accumulation, gliosis and cognitive impairment by blocking brain lymphatic drainage in APP/PS1 mice, which emphasizes the important role of MLVs in AD [72]. A recent study proposed that APOE4, a genetic risk factor for AD, functions in the shrinkage of MLVs and reduces the clearance of Aβ, ultimately leading to the progression of AD [85].

In contrast, many studies have demonstrated that boosting the function of MLVs could improve brain pathology in AD mouse models. For example, injection of recombinant VEGF-C, which promoted dural lymphangiogenesis, reduced the accumulation of Aβ and alleviated the cognitive deficits of APP/PS1 mice [73]. More recently, DSCR1, a Down syndrome-associated gene, was considered a regulator of MLVs function in AD. Indeed, the increase in MLVs drainage caused by DSCR1 overexpression diminished Aβ pathology in the brain and improved cognitive function in AD mouse models [86]. Additionally, Lin et al. demonstrated that repetitive transcranial magnetic stimulation (rTMS) treatment may reduce Aβ deposits by efficiently improving the drainage of Aβ via the meningeal lymphatics in 5xFAD brains [87].

In addition, the importance of MLVs function in improving AD pathology has also been demonstrated with immunotherapy. MLVs disturbance following treatment with Visudyne resulted in impaired CSF outflow and exacerbated AD symptoms, accompanied by a significant increase in macrophage numbers [14]. Furthermore, ablation of MLVs in 5xFAD mice that received anti-Aβ immunotherapy showed increased deposition of Aβ, while VEGF-C administration promoted the clearance of Aβ [73]. The numbers of CD4^+^ T cells and CD8^+^ T cells are increased significantly in 13 to 14 month-old 5xFAD mice, along with decreased MLVs coverage, but rescuing the function of MLVs with VEGF-C modified the efficacy of anti-Aβ immunotherapy and inhibited microgliosis, highlighting the potential of restoring MLVs function to improve neuroinflammation and cognitive function in AD [5, 88].

#### MLVs and the drainage of tau

Tau molecules belong to a family of microtubule-associated proteins that are predominantly found in neurons [89]. The intracellular accumulation of aggregated, hyperphosphorylated forms of tau in structures results in AD, so it is important to know what regulates the clearance of extracellular tau from the CNS.

It has been demonstrated that tau in the CSF can be transported into cervical lymph nodes through drainage of the MLVs in the dura [90]. Furthermore, Aspelund and Patel et al. reported that the dural lymphatic system also plays an important role in the clearance of extracellular tau using K14-VEGFR3-Ig transgenic mice, which lack functional dural lymphatics. Compared with that in WT mice, extracellular tau clearance is significantly impaired, and more tau is retained in the brains of K14-VEGFR3-Ig mice, which delays the clearance of tau into the plasma [3, 91]. Additionally, subarachnoid hemorrhage (SAH) increased the expression of brain tau and phosphorylated tau by impairing the drainage ability of MLVs [92].

Due to their pathophysiological role in clearing Aβ and Tau molecules, MLVs might be a promising therapeutic target for the prevention of AD.

### Meningeal lymphatics and PD

PD is an age-dependent neurodegenerative disease characterized by the loss of dopaminergic neurons in the midbrain and the formation of Lewy bodies [93, 94]. The accumulation and misfolding of α-synuclein protein are the main causes of PD; therefore, promoting the elimination of α-synuclein is the key step in the treatment of PD [95, 96]. Moreover, impairment of MLVs drainage has been shown to affect the development of PD pathology [97].

A53T mice, a mouse model of PD, subjected to ligation of the dCLNs, showed aggravated α-synuclein pathology compared to that of control mice. Disruption of meningeal lymphatic drainage via cervical lymphatic ligation promoted reactive gliosis and inflammatory cytokine production [97, 98]. In another study, mice injected with α-synuclein preformed fibrils to establish a model of PD exhibited decreased drainage from MLVs to the dCLNs, along with motor and memory deficits [6]. Importantly, the idea that the pathogenesis of PD is affected by MLVs was supported by evidence from human patients. MRI showed that CSF flow through the MLVs and dCLNs perfusion were decreased in idiopathic PD patients compared with atypical PD patients [6]. Additionally, MLVs dysfunction induced meningeal inflammation and meningeal lymphatic endothelial barrier dysfunction [6].

Therefore, the drainage of MLVs CSF is crucial for α-synuclein clearance, and dysfunction of MLVs could result in an abnormal meningeal immune response and contribute to neurodegenerative disease pathology.

### Meningeal lymphatics and brain tumors

Immune surveillance against tumors in the CNS is thought to be limited. However, recent studies have indicated that MLVs are also responsible for regulating the drainage and immune response of brain tumors, draining brain tumor cells from the CNS to dCLNs [7]. In GBM, DCs that carry antigen are trafficked to the dCLNs, where they can activate T cells. It has been reported that MLVs are involved in facilitating the trafficking of antigens, DCs and T cells to the dCLNs [99].

Song et al. reported that increasing lymphangiogenesis in the meninges with VEGF-C treatment promotes the migration of CD8^+^ T cells into the dCLNs and tumor site to evoke a long-lasting T-cell immune response and subsequently improves survival in a GBM mouse model. Importantly, delivery of VEGF-C has synergistic antitumor effects with checkpoint inhibitor therapy in a T-cell-dependent manner [60]. It is important to note that the combination of VEGF-C and immunotherapy can effectively reduce tumor growth, which is due to increased T-cell activation in the dCLNs and an immune response against the brain tumors [100]. Interestingly, brain tumors induce the remodeling of dorsal MLVs rather than basal MLVs; in turn, MLVs promote the spread of brain tumor cells and DCs to the dCLNs to stimulate specific T cells. For example, pharmacochemical ablation of dorsal MLVs blocked the spread of tumor cells from the brain to the dCLNs and reduced DCs traffic, thereby reducing the antitumor effect of anti-PD-1/CTLA-4 [7]. When anti-PD-1/CTLA-4 therapy was used to treat MLVs-ablated glioma mice, there was no increase in the antitumor immune response. Conversely, the efficacy of anti-PD-1/CTLA-4 was enhanced via CCL21/CCR7 signaling only when MLVs function was enhanced with VEGF-C. These results indicate that MLVs are the key to antitumor immunotherapy [7]. More recently, the same research group confirmed that the MLVs-dCLNs network affects the efficacy of radiotherapy for brain tumors. MLVs deficiency and dCLNs removal shortened overall survival and impaired radiotherapy-induced antitumor immunity, as DCs trafficking and CD8^+^ T-cell activation were inhibited [57]. VEGF-C overexpression, which stimulates meningeal lymphangiogenesis, improved the sensitivity of gliomas to radiotherapy in a CCL21-dependent manner [57].

Therefore, increasing immune cell trafficking by ameliorating the function of MLVs with VEGF-C will effectively enhance the efficacy of immunotherapy against brain tumors.

### Meningeal lymphatics and TBI

Mounting evidence indicates that TBI can cause impairments in cognition and mental health and is also a risk factor for the development of dementia, including AD [101, 102]. Moreover, TBI induces the secretion of proinflammatory cytokines and the infiltration of immune cells [103].

In mice, TBI induces loss of full MLVs function [8]. Bolte et al. found that meningeal lymphatic drainage function is impaired after mild TBI and that these deficits last 1 month post-injury. Mild head injury can impair CNS meningeal lymphatic function and induce changes in MLVs morphology, while existing MLVs dysfunction can lead to an increase in TBI-mediated neuroinflammation and cognitive impairment, such as an increase in Iba-1^+^ cell numbers and GFAP^+^ area [8]. Importantly, boosting MLVs function with VEGF-C after TBI decreases neuroinflammation in aged mice compared to mice that have not received VEGF-C [8]. A publication from Wojciechowski and colleagues showed that MLVs modulate the neuroimmune response during TBI. Although there was no difference in T lymphocytes between WT and K14-VEGFR3-Ig mice that completely lack lymphatics in several tissues, including the meninges, TBI impaired the CD4^+^ T-cell-mediated immune response in K14-VEGFR3-Ig mice, as demonstrated by a decrease in infiltrating CD4^+^ T cells, while the frequency of CD8^+^ T cells was increased, indicating that MLVs affect the impairment of the CD4^+^ T-cell-mediated neuroimmune response [104]. Another study showed that the accumulation of CD8^+^ T cells in the brains of mice after TBI led to neurological impairment [105].

In addition, subdural hematomas (SDHs) are hematomas that form between the arachnoid membrane and the dura mater. Liu et al. reported that autologous blood and A488-fibrinogen, components of SDHs, injected into the subdural space are drained into the dCLNs through MLVs, while ligation of deep cervical lymph vessels inhibits the clearance of SDHs. In turn, SDHs could also reduce the drainage ability of MLVs [106].

These results indicate that MLVs alleviate the inflammatory response by draining immune cells out of the brain. Thus, promoting remodeling of the meningeal lymphatic system may offer strategies to treat TBI patients.

### Meningeal lymphatics and MS

MLVs do not always play positive roles in neuroinflammation, and one such case is MS, an autoimmune disease [51]. MS is an autoimmune disease characterized by inflammatory demyelination of white matter in the CNS. In patients suffering from MS, the immune system destroys the myelin surrounding nerves [107]. The most widely used experimental mouse model of MS is EAE because it closely resembles the pathological features of MS [108]. Notably, the EAE model indicates that MLVs play important roles in the development and immune cell trafficking of MS [10, 51]. Without the full function of MLVs, there was an evident delay in the development of EAE and significantly milder disease pathology. However, meningeal lymphatic ablation can only attenuate and ameliorate EAE, suggesting that there are other routes involved [51].

Louveau et al. showed that the meningeal lymphatic system is crucial for the drainage and activation of brain-derived T cells in the dCLNs during the stimulation of neuroinflammation, while blocking MLVs via surgery or drugs decreased the inflammatory response of brain-reactive T cells in an animal model of MS and then ameliorated EAE pathology [51]. Since MLVs drain immune cells into the dCLNs in a CCR7-dependent manner, we believe that CCR7 is critical to inducing EAE. Therefore, intrathecal injection of CCR7^−/−^ T cells or DCs leads to their accumulation in the brain and failure to drain into the dCLNs [51, 109]. Consistent with these findings, transcriptomic analysis of MLVs-ablated mice showed that pathways associated with the inflammatory response and genes involved in T-cell activation and promoting EAE development were all downregulated [51]. Lymphatic vessels near the cribriform plate drain DCs to the dCLNs to maintain T-cell activation and increase the proliferation of reactive T cells in the dCLNs [10]. Additionally, LECs proliferate and undergo VEGFR3-dependent lymphangiogenesis in the meninges, so inhibition of lymphangiogenesis using MAZ51, a VEGFR3 tyrosine kinase inhibitor, resulted in a delay of EAE onset and reduced EAE severity [10].

Overall, MLVs may regulate the progression of MS by promoting T-cell activation and the inflammatory response, and ablation of MLVs could reduce the inflammatory response of T cells in MS.

### Meningeal lymphatics and stroke

It has been reported that stroke is the second leading cause of death [110]. Stroke includes ischemic stroke and hemorrhagic stroke, the latter of which is divided into intracerebral hemorrhage (ICH) and SAH [111, 112]. A recent study showed that MLVs play a neuroprotective role in stroke. They not only remove excess fluid from the brain but also grow into the injured parenchyma to eliminate edema [11].

With regard to ischemic stroke, meningeal lymphatic hypoplasia aggravates the severity of tMCAO in mice [113]. Similarly, ischemic stroke of zebrafish, induced by photothrombosis, promotes the ingrowth of MLVs into the injured parenchyma. The ingrown MLVs drain ISF to alleviate brain edema [114]. Interestingly, the drainage function of MLVs was significantly weakened in MCAO rats; in turn, meningeal lymphatic dysfunction aggravated neuroinflammation, neurological deficits and ischemic injury [115].

In addition, MLVs are also involved in ICH and SAH. Semyachkina-Glushkovskaya et al. verified that the meningeal lymphatics participate in brain clearance from the blood after ICH and that their diameter increases [116]. The MLVs also connect the ISF, CSF, and peripheral lymph [116]. Moreover, Tsai et al. found that ICH can promote meningeal lymphangiogenesis and enhance drainage function [117]. Ablation of MLVs or ligation of dCLNs hindered the clearance of hematomas, whereas enhancing the function of MLVs with VEGF-C reduced hematoma volume, accompanied by improved behavior, reduced neuronal loss and astroglia activation [117]. Chen and colleagues confirmed that erythrocytes are aggregated in the dCLNs and MLVs after SAH and that the MLVs are involved in the clearance of extravasated erythrocytes from the CSF into the dCLNs [118]. SAH persistently impairs the drainage ability of MLVs [92].

Thus, MLVs play a key role in stroke outcomes. Targeting MLVs is a potential approach for treating stroke.

## The relationship between MLVs and aging

Aging is associated with peripheral lymphatic dysfunction [119, 120], but the relationship between meningeal lymphatics and aging has rarely been reported. Recent studies suggested that aging is associated with both the dysfunction of MLVs and decreased drainage capacity of meningeal immune cells [9, 14]. For example, aging in mice induced a deleterious loss of meningeal lymphatic coverage and drainage capacity, which is closely associated with cognitive decline; notably, treatment of aged mice with VEGF-C could ameliorate this effect [14, 15]. Moreover, aging leads to reduced CCR7-expressing T cells in the meninges, along with changes in the transcriptional signature of meningeal LECs, and the abnormal meningeal immune response is accompanied by the further deposition of Aβ plaques [9]. Additionally, a clinical study showed that MLVs are impaired in the aging human brain through high-resolution T2-fluid attenuated inversion recovery imaging [69]. More excitingly, noninvasive MRI revealed age-related thickening of MLVs, and lymphatic output and waste removal were reduced in older subjects compared with younger subjects [12].

The aging process is a major risk factor for brain damage and even death. Chinta et al. found that glial cells are the predominant senescent cell type in the aging brain, and that senescent astrocytes damage adjacent neurons through secreting inflammatory factors [121]. It is worth noting that MLVs is an effective way to eliminate senescent astrocytes during aging process, and blocking meningeal lymphatic drainage impairs the clearance of senescent astrocytes in the brain parenchyma of aged mice, thereby increasing the neuroinflammatory response [55]. These results demonstrate the potential of MVLs as a therapeutic target for age-related CNS diseases.

## Conclusions and perspectives

**Fig. 4:**
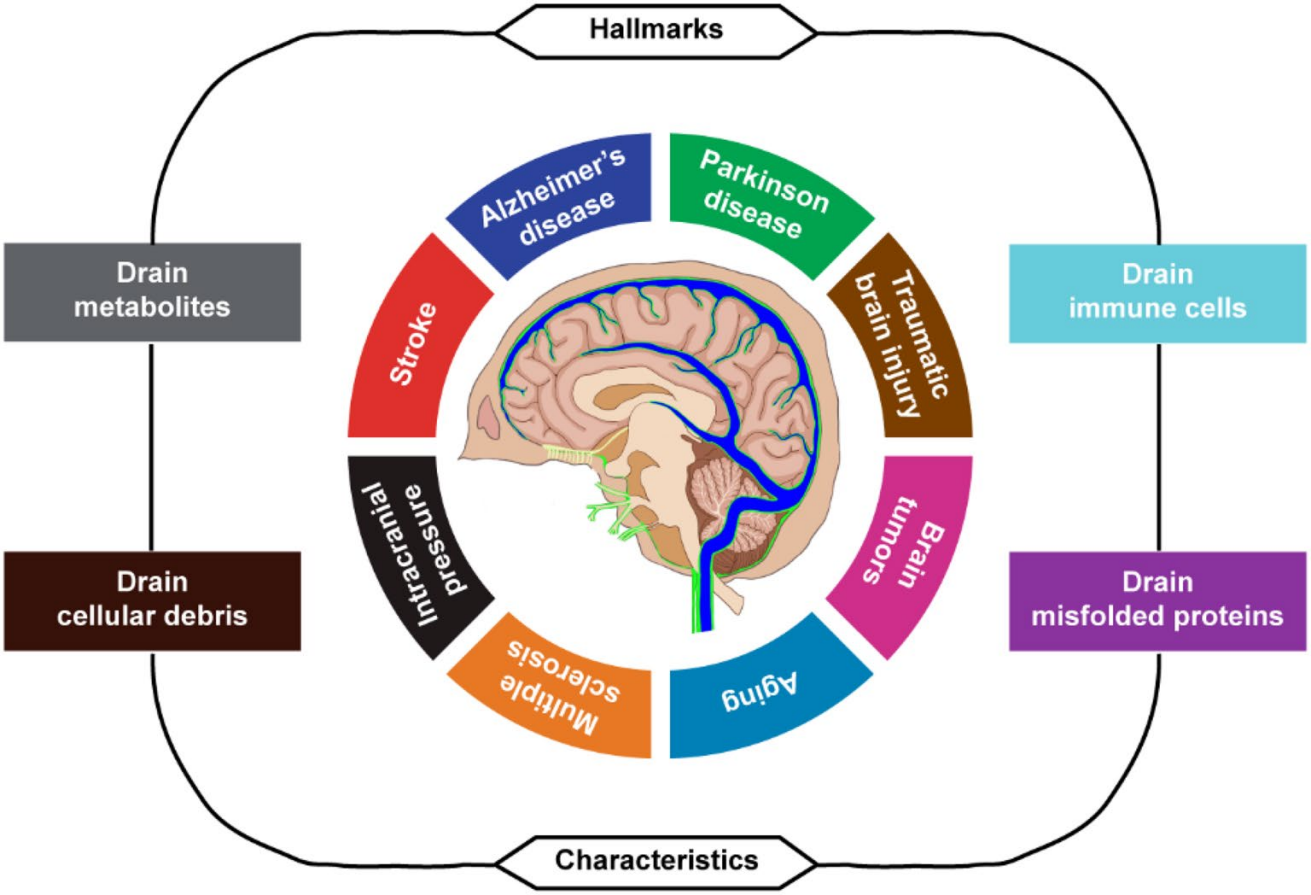
Physiopathological hallmarks of MLVs. This illustration encompasses eight hallmark capabilities of MLVs. We have witnessed remarkable progress in our knowledge of MLVs. In the future, the mechanistic underpinnings of each hallmark will be well understood

Since the rediscovery of MLVs in 2015, many studies have confirmed the importance of MLVs in neurological disorders and aging (Fig. 4). The meningeal lymphatics interact with other clearance mechanisms to maintain homeostasis of the brain by draining metabolites, cellular debris, misfolded proteins, and immune cells shuttle between the brain and the periphery. Finally, understanding the function of MLVs in regulating lymphatic drainage may provide therapeutic targets to treat neurological diseases even delay brain aging process of humans.

## Data Availability

All data are included in the manuscript.

## Abbreviations

CNS: Central nervous system
MLVS: Meningeal lymphatic vessels
CSF: Cerebrospinal fluid
ISF: Interstitial fluid
dCLNs: Deep cervical lymph nodes
AD: Alzheimer’s disease
PD: Parkinson’s disease
TBI: Traumatic brain injury
MS: Multiple sclerosis
SAS: Subarachnoid space
MRI: Magnetic resonance imaging
LECs: Lymphatic endothelial cells
Prox1: Proprospero homeobox protein 1
Lyve-1: Lymphatic endothelial hyaluronic acid receptor 1
PDPN: Podoplanin
FGF-2: Fibroblast growth factor 2
IGF-1: Insulin-like growth factor 1
HGF: Hepatocyte growth factor
ET-1: Endothelin-1
SMCs: Smooth muscle cells
APP: β-Amyloid precursor protein
rTMS: Repetitive transcranial magnetic stimulation
SAH: Subarachnoid hemorrhage
GBM: Glioblastoma multiforme
SDHs: Subdural hematomas
EAE: Experimental autoimmune encephalomyelitis
ICH: Intracerebral hemorrhage
SSS: Superior sagittal sinus
TS: Transverse sinus
APCs: Antigen-presenting cells
DCs: Dendritic cells
AQP-4: Aquaporin-4
